# Constitution of mucosa‐associated microbiota in the lower digestive tract does not change in early stage of non‐alcoholic fatty liver disease with fecal dysbiosis

**DOI:** 10.1002/jgh3.12803

**Published:** 2022-08-29

**Authors:** Naoki Asaji, Jun Inoue, Hiroki Hayashi, Eri Tokunaga, Yusaku Shimamoto, Masato Kinoshita, Takeshi Tanaka, Arata Sakai, Yoshihiko Yano, Yoshihide Ueda, Yuzo Kodama

**Affiliations:** ^1^ Department of Internal Medicine, Division of Gastroenterology Kobe University Graduate School of Medicine Kobe Japan; ^2^ Division of Gastroenterology Kita‐Harima Medical Center Ono Japan

**Keywords:** 16S rDNA, dysbiosis, mucosa‐associated microbiota, non‐alcoholic fatty liver disease

## Abstract

**Background and Aim:**

Regarding the gut–liver axis, fecal dysbiosis is implicated in the pathogenesis of non‐alcoholic fatty liver disease (NAFLD). The significance of mucosa‐associated microbiota (MAM, which is present in the mucin layer covering the intestinal mucosa) has not been well explored. We aimed to clarify the characteristics of MAM in patients with NAFLD.

**Methods:**

MAM were obtained from seven patients with early‐stage NAFLD and seven controls by colonoscopy in five locations (terminal ileum, cecum, ascending and sigmoid colon, and rectum) using mucosal brushes. The microbial 16S rDNA profiles of the MAM and fecal microbiota of patients in the NAFLD and control groups were analyzed.

**Results:**

α‐diversities of fecal microbiota were decreased in patients with NAFLD (observed species, Shannon index, and Chao1: 174.57 *vs* 134.86, 5.51 *vs* 4.65, and 206.34 *vs* 167.91; *P* = 0.048, 0.067, and 0.087, respectively), and microbial composition analyses by principal coordinate analysis differed between the fecal microbiota of patients with NAFLD and those of controls (permutational analysis of variance [PERMANOVA] of weighted and unweighted: Pseud‐*F*: 1.4179/*P*‐value: 0.05 and Pseud‐*F*: 2.1497/*P*‐value: 0.049, respectively). However, α‐diversities or microbial composition of MAM in most parts of the intestine did not differ significantly between the NAFLD and control groups. Unclassified *Rikenellaceae*, Oscillospira, Odoribacter, unclassified *clostridiales*, and *Holdemania* were decreased in the feces of patients with NAFLD (determined by linear discriminant analysis effect size), but five (except *Holdemania*) of the six genera were not decreased in the MAM of these patients.

**Conclusion:**

In early‐stage NAFLD, MAM was uniform and relatively stable throughout the intestine, even when fecal dysbiosis appeared.

## Graphical abstract

The α‐diversities of fecal microbiota were decreased in patients with non‐alcoholic fatty liver disease (NAFLD), and microbial composition by principal coordinate analysis differed between the fecal microbiota of patients with NAFLD and those of controls. However, α‐diversities or microbial composition of mucosa‐associated microbiota (MAM) in most parts of the lower gut (from the terminal ileum to the rectum) did not differ between the NAFLD and control groups. In early‐stage NAFLD, MAM was uniform and relatively stable throughout the intestine, even when fecal dysbiosis appeared.

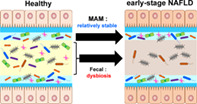

## Introduction

Non‐alcoholic fatty liver disease (NAFLD) is currently the leading cause of chronic liver disease in developed countries, and the number of patients with NAFLD is increasing worldwide.^1^, ^2^, ^3^ The influx of pathogen‐associated molecular patterns (PAMPs) into the portal vein due to increased intestinal permeability is thought to be part of the pathophysiology of NAFLD, and the bidirectional relationship between the gastrointestinal tract and liver (the gut–liver axis) is considered important.^4^ Gut dysbiosis increases insulin resistance and intestinal permeability, which promotes oxidative stress and chronic exposure of PAMPs.^5^ Gut dysbiosis is reportedly present in patients with NAFLD.^6^, ^7^, ^8^ Intestinal microbiota are distinguished by their location as follows: lumen‐associated microbiota (present in intestinal contents) and mucosa‐associated microbiota (MAM; present in the mucin layer). The composition of bacteria in MAM is significantly different from that of bacteria in lumen‐associated microbiota such as those in feces.^9^ Since a change in MAM is associated with intestinal epithelial damage and increased intestinal permeability,^10^, ^11^ it is important to analyze MAM when considering the effects of the gut microbiota on a pathology. However, although the fecal microbiota of patients with NAFLD, which are the final form of lumen‐associated microbiota, have been well‐analyzed, the characteristics of MAM that could potentially affect mucosal permeability have not been analyzed. This study aimed to characterize the MAM in patients with NAFLD and analyze the association between MAM and NAFLD. MAM (obtained by endoscopy) and fecal microbiota were subjected to 16S rDNA sequencing analysis using a next‐generation sequencer for comparisons between individuals in the NAFLD and control groups.

## Methods

### Patient cohorts

Patients with and without (control) NAFLD who were age‐ and gender‐matched using propensity score (ratio 1:1, caliper 0.2) were enrolled in this study between 1 June 2017, and 1 March 2019 in our hospital. This study was conducted according to the guidelines of Clinical Research Ethical Committee, Kobe University Graduate School of Medicine. This study was approved by Kobe University Graduate School of Medicine (Clinical Research Ethical Committee No. 170043, B200357). NAFLD was diagnosed based on liver biopsy or imaging (abdominal ultrasonography or computed tomography), and those with fatty liver resulting from the consumption of alcohol or drugs or due to genetic disorders were excluded.^12^, ^13^ Patients without NAFLD who did not use medications (within 1 month prior to the study) that could potentially affect the fecal microbiota (including probiotics or antibiotics) comprised the control group. Propensity score matching was performed using EZR software (Easy R version 1.54, Saitama Medical Center, Jichi Medical University, Japan), and the control and NAFLD groups comprised seven patients each.

### Sample collection

Samples of intestinal mucus and feces were collected for gut microbiota analysis. Fecal sample from the first stool passed on the day of the examination was collected in a sterile bottle and immediately cooled in a box with ice. During colonoscopy, mucus samples were obtained by scraping the mucous membranes of the terminal ileum, cecum, transverse and sigmoid colon, and rectum with a disposable cytology brush (BC‐23Q®); they were then diluted in phosphate‐buffered saline (PBS) and stored at −80°C.

### DNA extraction

Bacterial DNA was extracted from 70 to 00 mg of each fecal sample and from 1 mL of each mucus sample contained in PBS. After centrifuging the saliva and mucus samples for 10 min at 13000×g and 4°C, the supernatant was decanted and disposed of, while the precipitate was retained. DNA extraction from the samples was performed using a QIAamp PowerFecal DNA Kit (Qiagen, Manchester, UK) according to the manufacturer's protocol. Each sample was stored at −20°C.

### 16S rDNA sequencing

The V3–V4 regions of the gene encoding 16S rDNA were amplified using the two‐step thermal asymmetric interlaced polymerase chain reaction (TAIL‐PCR) method, and 16S rDNA sequencing was performed using the MiSeq™ system (Illumina, San Diego, CA, USA) according to the manufacturer's protocol, with a slight modification. In brief, for the first step of PCR, DNA extracted from mucus and stool samples was amplified using Amplicon PCR primers and 2x KAPA HiFi HotStart ReadyMix (KAPA Biosystems, KK2602, Woburn, MA, USA) and then purified with AMPure XP Beads (Beckman Coulter, A63881, San Diego, CA, USA) and 80% ethanol. The sequences of the forward and reverse primers with overhang adapters were 5‐TCGTCGGCAGCGTCAGATGTGTATAAGAGACAG‐3 and 5‐GTCTCGTGGGCTCGGAGATGTGTATAAGAGACAG‐3, respectively. For the second step of PCR, the purified amplicon was further amplified using Nextera XT Index Primer (Illumina, FC‐131‐2001, San Diego, CA, USA), 2x KAPA HiFi HotStart ReadyMix, and sterile, purified water. DNA was purified using AMPure XP Beads and 80% ethanol and then further purified with SPRIselect Beads (Beckman Coulter, B23317, Brea, CA, USA) and 85% ethanol to remove any non‐specifically amplified large DNA fragments. DNA concentration was measured using a Qubit® 2.0 Fluorometer (Invitrogen, Q33216) and TapeStation (Agilent Technologies, 5067–5584/5585). Pooled samples were sequenced on the MiSeq system using a MiSeq Reagent Kit v2 (500 cycles; Illumina, San Diego, CA, USA).

### Microbiome analysis of 16S rDNA sequencing

The QIIME (version 1.9.0) pipeline was used to perform sequence read processing, quality trimming, operational taxonomic unit (OTU) definition, and taxonomic assignments. The 3′‐end low‐quality bases were trimmed using the PReprocessing and INformation of SEQuences (PRINSEQ) tool (version 0.20.4.),^14^ and paired‐end reads were merged using the fastq‐join program. Chimeric sequences were removed using USEARCH 6.1.544. OTU clustering was performed using UCLUST at a justification for the 97% threshold.^15^ The sequence reads were then searched in the Greengenes reference database (version 13.8).^16^ All OTUs were classified into bacterial groups at the genus level based on 97% sequence similarity against the Greengenes database (gg_13_8). The alpha (α)‐diversity, including the following indices, was calculated using QIIME: the Shannon index, observed species, and Chao1 index. Weighted and unweighted UniFrac principal coordinate analysis (PCoA) and permutational analysis of variance (PERMANOVA) were performed using QIIME. Linear discriminant analysis (LDA) effect size (LEfSe) calculations were performed to estimate the effect size of each taxon with significant differential abundance using Galaxy Version 1.0 (The Huttenhower Lab, Harvard T.H. Chan School of Public Health).

### Statistical analyses

α‐diversity indices (observed species, Shannon index, and Chao1 index) were analyzed using Student's *t*‐tests. Beta (β)‐diversity was estimated using the UniFrac distance metric and analyzed using the chi square test or PERMANOVA. A *P*‐value ≤0.05 was considered to indicate statistical significance.

## Results

### Clinical characteristics of the study participants

Compared with the control group, the body mass index, aspartate aminotransferase, alanine aminotransferase, gamma‐glutamyl transpeptidase, and total protein values were significantly higher in the NAFLD group, whereas other parameters, such as age, sex, and Fib4‐index, did not differ significantly (Table 1).

**Table 1:** Clinical characteristics of the study participants

Characteristic	Control (*n* = 7)	NAFLD (*n* = 7)	*P*‐value
Sex (male/female)	4/3	5/2	0.58
Age (years)	64.7 (7.7)	63.6 (8.4)	0.81
Height (cm)	164.5 (9.9)	163.0 (10.0)	0.80
Weight (kg)	59.3 (9.5)	67.1 (11.7)	0.22
BMI (kg/m^2^)	21.8 (1.6)	25.1 (2.8)	**0.023**
AST (U/L)	19 (4)	36 (11)	**0.004**
ALT (U/L)	16 (6)	42 (13)	**0.001**
ALP (U/L)	225 (32)	251 (47)	0.28
γGTP (U/L)	22 (7)	41 (11)	**0.005**
PLT (10^9^/L)	197 (38)	207 (55)	0.726
TP (g/dL)	7.0 (0.4)	7.7 (0.4)	**0.006**
ALB (g/dL)	4.4 (0.3)	4.7 (0.2)	0.087
T‐Bil (mg/dL)	0.9 (0.4)	0.9 (0.2)	0.938
T‐CHOL (mg/dL)	214 (23)	213 (44)	0.956
HDL‐CHOL (mg/dL)	68 (24)	62 (11)	0.626
TG (mg/dL)	110 (38)	110 (28)	0.983
Glucose (mg/dL)	100 (7)	118 (22)	0.082
FIB‐4 index	1.64 (0.53)	2.29 (2.42)	0.535

*P*‐values with significant difference between the two groups are in bold.

Values for sex are expressed as the number of individuals; for all other characteristics, the values are expressed as mean (SD). The values of BMI, AST, ALT, γGTP, and TP were significantly higher in the NAFLD group compared with the healthy controls (Student's *t*‐test, *P* < 0.05).

ALB, albumin; ALP, alkaline phosphatase; ALT, alanine aminotransferase; AST, aspartate aminotransferase; BMI, body mass index; LDL‐CHOL, low‐density lipoprotein cholesterol; NAFLD, non‐alcoholic fatty liver disease; PLT, platelets; T‐Bil, total bilirubin; T‐CHOL, total cholesterol; TG, triglycerides; TP, total protein; γGTP, gamma‐glutamyl transpeptidase.

### Alteration of fecal microbiome in NAFLD

**Figure 1:**
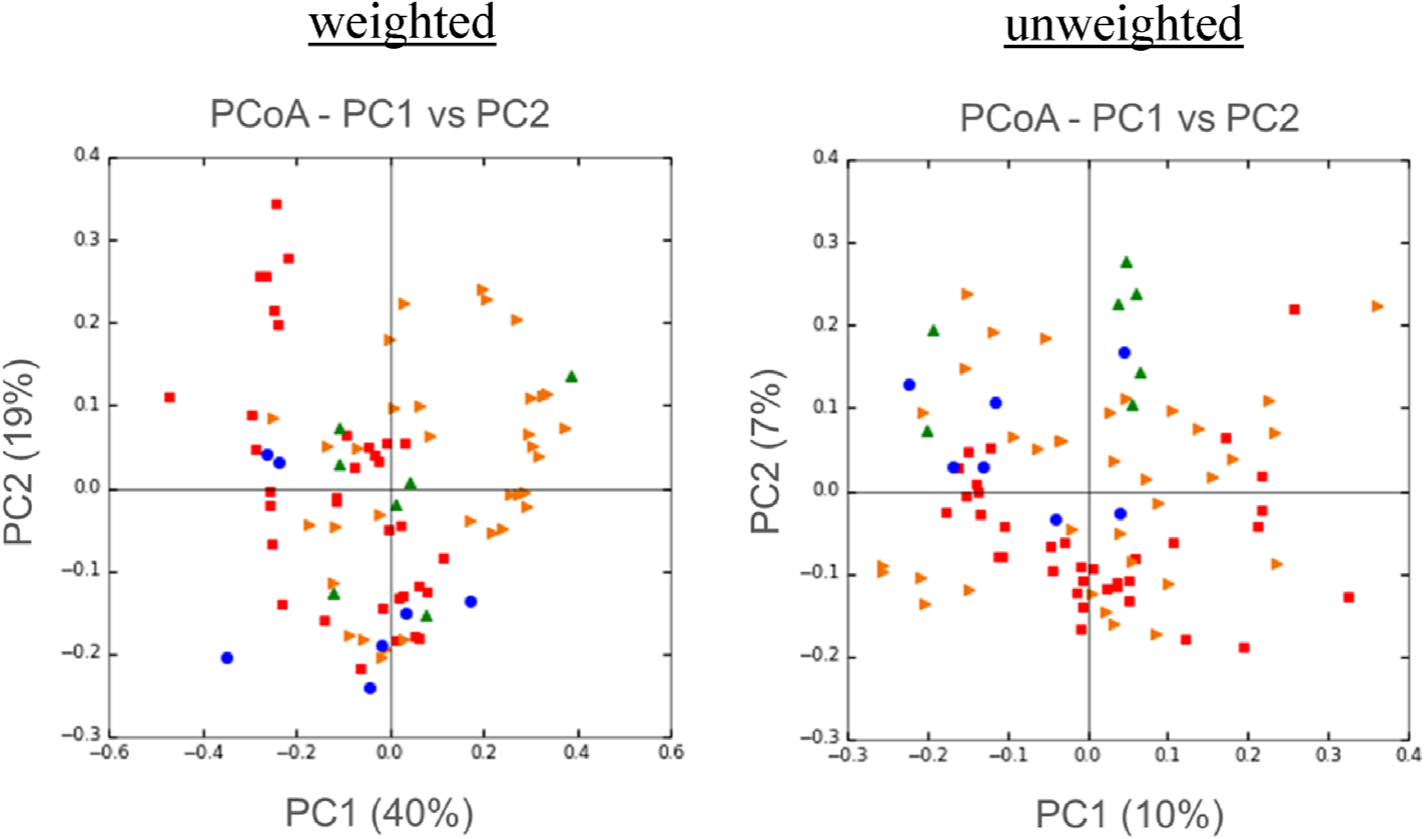
Principal coordinate analysis plots of the microbial community in fecal and mucosa‐associated (MAM) microbiota in all patients with weighted and unweighted UniFrac distance. Each plot represents an individual sample. The fecal samples of patients in the control and non‐alcoholic fatty liver disease (NAFLD) groups (*n* = 7/group) are represented by blue and green plots, respectively. The mucosal samples of patients in the control and NAFLD groups (*n* = 35/group) are represented by red and orange plots, respectively. 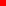, Control (MAM); 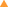, NAFLD (MAM); 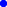, control (feces); 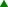, NAFLD (feces).

**Figure 2:**
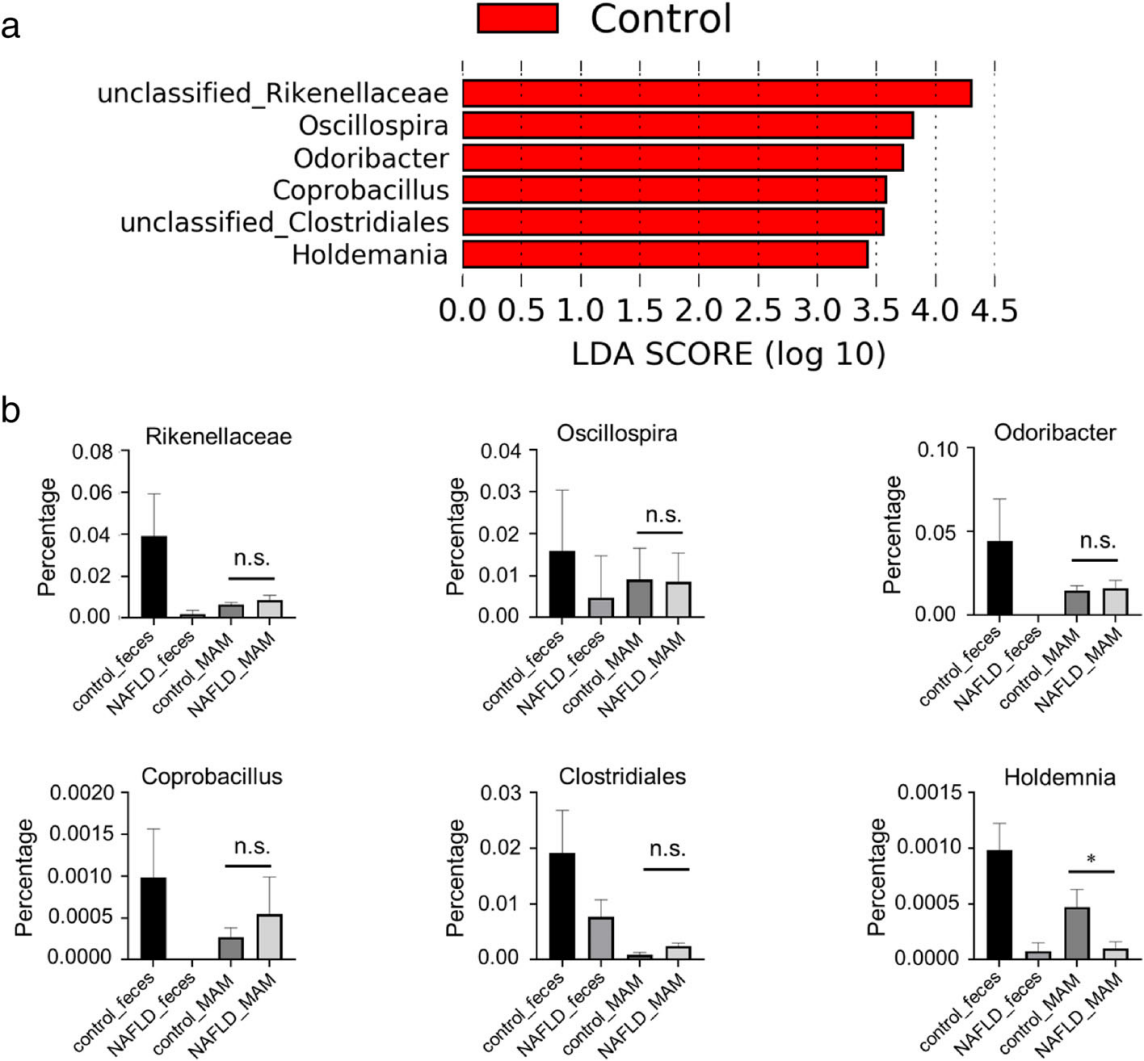
Changes in the fecal bacterial flora of patients with non‐alcoholic fatty liver disease (NAFLD) and the relative amount of bacteria in mucosa‐associated microbiota (MAM). (a) LEfSe calculations between patients in the control and NAFLD groups are performed using a threshold of 2.0 and an alpha threshold of 0.05 to identify the taxa at baseline. Six genera are significantly decreased in the fecal samples of patients with NAFLD compared to the control group. (b) Comparison of the proportion of microbes in the MAM that had decreased in the feces between patients with NAFLD and those without (controls). Five of the six genera that significantly decreased in the fecal samples of patients with NAFLD are not decreased in their MAM. *P*‐values were evaluated using the one‐way analysis of variance test. *P*‐value ≤0.05 was considered to indicate statistical significance. *P < 0.01; LEfSe, Linear discriminant analysis effect size; n.s., not significant.

α‐diversity reduction and compositional changes have been observed in the fecal microbiota of patients with NAFLD.^6^, ^7^, ^8^, ^17^, ^18^, ^19^ First, we analyzed the α‐diversity and microbial composition of fecal samples and compared them between patients in the NAFLD and control groups. Images of microbial structure with PCoA in all NAFLD and control samples are shown in Figure 1. In the analysis of α‐diversity, the observed species was significantly decreased in the fecal microbiota of patients with NAFLD and a tendency of a reduction in the Shannon and Chao1 indices was also observed (*P* = 0.048, 0.067, and 0.087; Table 2). Next, to determine whether there was a compositional change in the fecal microbiota of patients with NAFLD, we analyzed the β‐diversity of weighted and unweighted PCoA in their fecal microbiota and in those patients in the control group. PERMANOVA in both weighted and unweighted PCoA suggested that there were compositional microbial changes in the fecal microbiota of patients in the NAFLD and control groups (Pseud‐*F*: 1.4179/*P*‐value: 0.05; Pseud‐*F*: 2.1497/*P*‐value: 0.049, respectively; Table 2). These results indicate that there is dysbiosis of the fecal microbiota of patients with NAFLD in our study. In the fecal microbiota of patients with NAFLD, LEfSe analysis indicated that compositional changes were characterized by a significant decrease in the abundance of six genera of bacteria including unclassified Rikenellaceae, Oscillospira, Odoribacter, unclassified clostridiales, and Holdemania (Fig. 2a).

**Table 2:** Alpha‐diversity and PERMANOVA for the comparisons of the microbial communities between healthy controls and patients with NAFLD

	alpha‐diversity (Control vs NAFLD)		PERMANOVA of PCoA (Control vs NAFLD)	
	Observed species Shannon Chao 1	p‐value	Pseudo‐f of weighted and unweighted	p‐value
Fecal microbiota	174.57 ± 29.36 vs 134.86 ± 32.92	0.048 *	1.4179 2.1497	**0.05** * **0.049** *
	5.51 ± 0.51 vs 4.65 ± 0.92	0.067		
	206.34 ± 31.55 vs 167.91 ± 39.40	0.087		
MAM				
Terminal ileum	138.57 ± 28.11 vs 124.57 ± 16.69	0.315	2.4528 0.8406	0.065 0.813
	5.25 ± 0.33 vs 4.71 ± 0.31	0.013 *		
	178.85 ± 41.14 vs 149.34 ± 19.53	0.138		
Cecum	156.29 ± 16.93 vs 145.29 ± 34.73	0.499	1.0289 0.8836	0.39 0.718
	5.21 ± 0.44 vs 5.00 ± 1.00	0.652		
	205.87 ± 31.46 vs 188.18 ± 49.68	0.475		
Transverse colon	147.29 ± 19.31 vs 134.43 ± 40.01	0.492	2.0392 0.7977	0.058 0.944
	4.92 ± 0.76 vs 4.72 ± 1.18	0.730		
	193.01 ± 16.67 vs 167.44 ± 49.39	0.253		
Sigmoid colon	131.86 ± 33.82 vs 121 ± 39.09	0.616	2.5625 1.1585	**0.05** * 0.196
	4.70 ± 0.97 vs 4.88 ± 0.82	0.727		
	164.04 ± 50.15 vs 149.02 ± 43.90	0.591		
Rectum	143.14 ± 42.21 vs 140.71 ± 38.38	0.919	1.7415 0.9133	0.089 0.656
	4.83 ± 1.09 vs 5.29 ± 0.63	0.395		
	176.81 ± 63.27 vs 172.00 ± 46.18	0.883		

*P*‐values with significant difference between the two groups are in bold.

* *P* ≤ 0.05.

The results of the α‐diversity (mean,  ± SD) and PERMANOVA for bacterial community profiles.

MAM, mucosa‐associated microbiota; NAFLD, non‐alcoholic fatty liver disease; PERMANOVA, permutational analysis of variance.

### The state of MAM in patients with NAFLD in whom dysbiosis of fecal microbiome has occurred

To confirm whether there were microbial changes in the MAM of patients with NAFLD in whom dysbiosis of fecal microbiome had occurred, we analyzed the α‐diversity and microbial composition of MAM in patients in the control and NAFLD groups. MAM at each anatomical site were compared between the patients in the two groups. Contrary to expectations, no significant difference was observed in the α‐diversity of MAM in all anatomical sites between patients with NAFLD and those in the control group, except for a decrease in Shannon index of the terminal ileum in patients with NAFLD (Table 2). Microbial composition analysis with weighted and unweighted PCoA also revealed that despite the difference in fecal microbiota between patients in the NAFLD and control groups, MAM showed no significant differences in weighted and unweighted PCoA, except in weighted PCoA of the sigmoid colon (Table 2). These results suggest that MAM may be relatively stable even in the presence of fecal microbial change in NAFLD. Furthermore, among the six microbes that had decreased in the feces of patients with NAFLD (observed by LEfSe analysis), five were retained in their MAM (Fig. 2b).

### The intra‐ and inter‐individual MAM structure in patients with NAFLD was similar to that of individuals in the control group

**Figure 3:**
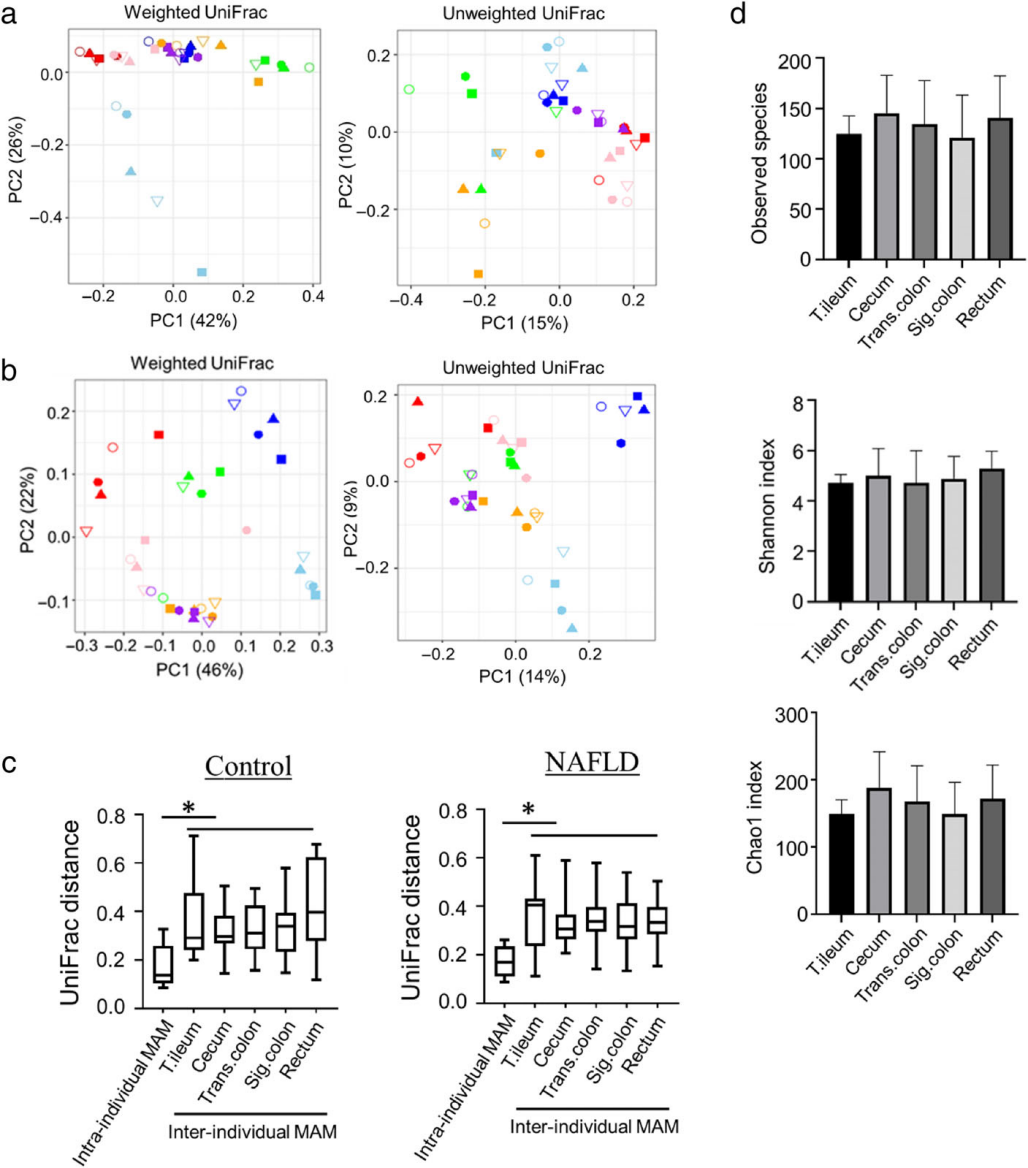
The intra‐individual mucosa‐associated microbiota (MAM) were similar than inter‐individual MAM both in patients with non‐alcoholic fatty liver disease (NAFLD) and in the control group. (a,b) Weighted principal coordinate analysis (PCoA) plots of beta‐diversity measures of the composition of MAM in the control (a: 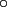, terminal ileum; 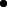, cecum; 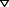, tranverse colon; 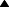, sigmoid colon; 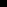, rectum; 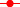, control 1; 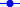, control 2; 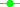, control 3; 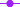, control 4; 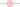, control 5; 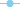, control 6; 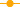, control 7) and NAFLD (b: 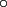, terminal ileum; 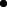, cecum; 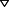, tranverse colon; 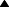, sigmoid colon; 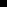, rectum; 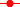, NAFLD 1; 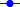, NAFLD 2; 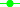, NAFLD 3; 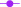, NAFLD 4; 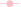, NAFLD 5; 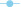, NAFLD 6; 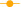, NAFLD 7) groups. Each plot represents an individual sample. The form of each plot is different for each anatomical site, and the color is different for each individual. There is no significant difference by permutational analysis of variance (PERMANOVA) among the different anatomical sites. (c) Comparison of weighted UniFrac distance of intra‐individual MAM and inter‐individual MAM in each anatomical site. In both groups, the intra‐individual MAM at each site are more similar to the inter‐individual MAM at the same anatomical site. (d) Comparative analysis of the α‐diversity of the microbiota communities between each MAM in patients with NAFLD. There are no significant differences in α‐diversity of MAM among the different anatomical sites. *P*‐values were evaluated using Dunnett's test (*P < 0.05). sig. colon, sigmoid colon; T. Ileum, terminal ileum; trans. colon, transverse colon.

The composition of MAM in the lower gut (from the terminal ileum to the rectum) is similar in healthy individuals.^20^ We first confirmed the similarity of MAM structure in the different anatomical sites in patients in the NAFLD and control groups using β‐diversity constructed with weighted and unweighted UniFrac distance. In the control group, PCoA showed that there were no significant differences in microbial structure among the anatomical sites (terminal ileum, cecum, transverse colon, sigmoid colon, and rectum; *P*‐values of PERMANOVA in weighted and unweighted PCoA were 1.0 and 0.996, respectively; Fig. 3a); this finding is comparable to that of a previous study.^20^ Similarly, in patients with NAFLD, there were no significant differences in microbial structure among the anatomical sites (*P*‐values of PERMANOVA in weighted and unweighted PCoA were 0.996 and 1.0, respectively; Fig. 3b). These results indicate that MAM in the lower gut (from the terminal ileum to the rectum) are similar with or without NAFLD. However, samples from the same individual were located closer together than samples from different individuals in both study groups (Fig. 3c), suggesting that the intra‐individual MAM at different sites are more similar than the inter‐individual MAM.

We also compared the α‐diversity of MAM among the different anatomical sites using three indices: the observed species and Chao1 index, which reflect OTU richness, and the Shannon index, which reflects OTU evenness. There was no statistically significant difference in α‐diversity among the five anatomical sites (terminal ileum, cecum, transverse colon, sigmoid colon, and rectum) in individuals in the control group (data not shown). Similarly, in patients with NALFD, there were no significant differences in α‐diversity of MAM among the different sites (Fig. 3d). These results indicate that the composition and α‐diversity of MAM in patients with NAFLD were similar among the five anatomical sites.

## Discussion

NAFLD is a phenotype of metabolic syndrome in the liver; its incidence has been increasing worldwide in recent years. Fecal dysbiosis is reportedly involved in the pathogenesis of many diseases related to metabolic syndrome. Since intestinal bacteria directly reach the liver through the portal circulation, dysbiosis of the intestinal microbiota is also greatly involved in the onset and progression of NAFLD. The bidirectional relationship between the gut (including microbiota) and liver is now well‐recognized as the gut–liver axis, and a disruption of the intestinal mucosal barrier affects the homeostasis of this axis. This study also supported the involvement of fecal dysbiosis in the pathogenesis of NAFLD and revealed that several types of bacteria were decreased in the feces of patients with NAFLD (characterized by LEfSe).

In contrast, the gut microbiota consists of the luminal microbiota (including fecal microbiota) and MAM (present in the intestinal mucosa).^9^ MAM reflects mucosal barrier function more directly than fecal microbiota and is reportedly involved in the pathogenesis of many diseases, such as intestinal diseases like inflammatory bowel disease and extraintestinal diseases including liver disease.^21^, ^22^, ^23^, ^24^, ^25^ The overgrowth of MAM is associated with the pathogenesis of primary biliary cirrhosis.^26^ This study analyzed the characteristics of MAM in early‐stage NAFLD and the differences between fecal microbiota and MAM. We found that the microbes in the MAM of the lower gut of patients with NAFLD were not changed even though there were microbial changes in their feces. To the best of our knowledge, this is the first study to analyze the human MAM from the terminal ileum to the rectum (obtained using endoscopy) in patients with NAFLD; the results of our analysis could be important when considering the involvement of the gut microbiota in the pathophysiology of NAFLD.

First, we performed a comparative analysis of MAM and fecal microbiota between patients in the NAFLD and control groups. Similar to previous reports,^6^, ^7^, ^8^ reduced α‐diversity and changes in fecal microbial composition in patients with NAFLD were observed in our study. However, in contrast to the fecal microbial changes in our study, the reduced α‐diversity or microbial compositional change observed in the MAM of patients with NAFLD in this study was limited. Furthermore, among the six microbes that were decreased in patients with NAFLD (characterized by LEfSe), five were retained in their MAM. These results may be due to the relative stability of MAM in patients with NAFLD. There may be environmental factors that stabilize MAM in the mucous layer even in the presence of changes in the intestinal environment, such as diet change. One dominant factor may be the influx of glycans to the surface of the intestine. In contrast to diet‐derived glycans that vary in composition and supply, host‐derived glycans from the mucus layer provide a more continuous source of certain constituent nutrients for the microbes, which utilize mucin as their nutrient source.^27^ In addition, immunoglobulin A (IgA) is secreted and anchored to the mucus layer, exhibiting low‐affinity polyreactive binding to numerous microbial antigens, which enables colonization and elimination of intestinal bacteria in the mucus.^28^, ^29^, ^30^ Due to the continuous supply of mucus and IgA, MAM may exist in a stable system. In fact, in diseases such as inflammatory bowel disease and irritable bowel syndrome, in which changes in both fecal microbial flora and MAM have been reported,^11^, ^13^ changes in mucin levels and the quality of secreted IgA have been reported.^31^, ^32^, ^33^ Regarding the pathophysiology of early‐stage NAFLD, changes in metabolites due to dysbiosis of the intraluminal microbiota (as observed in feces) may be more strongly involved than the direct effects of changes in MAM on the mucosa.

Next, we examined the differences in MAM by site. Although the α‐diversity or microbial composition of MAM from the rectum to the ileum is homogeneous in healthy individuals,^20^ our study revealed that no site‐specific differences in α‐diversity or microbial composition of MAM were observed in patients with NAFLD. These results also suggest that microbial structures in the MAM of patients with early‐stage NAFLD are not different from those in the MAM of controls.

This study has some limitations. First, the sample size was limited in this study. Second, the cases of NAFLD enrolled in this study were relatively early stage (the average Fib4‐index of the NAFLD group was 2.29). Intestinal permeability is enhanced in advanced‐stage NAFLD,^34^ and it is possible that changes in the MAM of patients with advanced NAFLD may be visible. However, our study confirms that MAM is relatively more stable than fecal microbiota in early‐stage NAFLD.

## Conclusions

To the best of our knowledge, this is the first study in humans to investigate the MAM of different anatomical sites (obtained during endoscopy) in the lower gut of patients with NAFLD. In early‐stage NAFLD, MAM was uniform and relatively stable throughout the intestine, even when fecal dysbiosis appeared. This study can be a source of basic knowledge for studying the involvement of the gut microbiota in the pathophysiology of NAFLD.

## Consent for Publication

All subjects provided written informed consent to participate prior to beginning the study. No subjects offered refusal for publication.

## Data Availability

The data used in the study's analyses are not available to the public. Sequence reads of samples used in this study were deposited in the DNA Data Bank of Japan Sequence Read Archive (http://www.ddbj.nig.ac.jp/index-e.html) under accession number DRA013738.
